# Genetic Variability of the mTOR Pathway and Prostate Cancer Risk in the European Prospective Investigation on Cancer (EPIC)

**DOI:** 10.1371/journal.pone.0016914

**Published:** 2011-02-23

**Authors:** Daniele Campa, Anika Hüsing, Angelika Stein, Lucie Dostal, Heiner Boeing, Tobias Pischon, Anne Tjønneland, Nina Roswall, Kim Overvad, Jane Nautrup Østergaard, Laudina Rodríguez, Núria Sala, Maria-José Sánchez, Nerea Larrañaga, José María Huerta, Aurelio Barricarte, Kay-Tee Khaw, Nicholas Wareham, Ruth C. Travis, Naomi E. Allen, Pagona Lagiou, Antonia Trichopoulou, Dimitrios Trichopoulos, Domenico Palli, Sabina Sieri, Rosario Tumino, Carlotta Sacerdote, Henk van Kranen, H. Bas Bueno-de-Mesquita, Göran Hallmans, Mattias Johansson, Isabelle Romieu, Mazda Jenab, David G. Cox, Afshan Siddiq, Elio Riboli, Federico Canzian, Rudolf Kaaks

**Affiliations:** 1 German Cancer Research Center (DKFZ), Heidelberg, Germany; 2 Department of Epidemiology, Deutsches Institut für Ernährungsforschung, Potsdam-Rehbrücke, Germany; 3 The Danish Cancer Society, Institute of Cancer Epidemiology, Copenhagen, Denmark; 4 Department of Cardiology, Center for Cardiovascular Research, Aalborg Hospital, Aarhus University Hospital, Aalborg, Denmark; 5 Department of Epidemiology, School of Public Health, Aarhus University, Denmark; 6 Public Health and Participation Directorate, Health and Health Care Services Council, Asturias, Spain; 7 Catalan Institute of Oncology (ICO) - IDIBELL, Barcelona, Spain; 8 Andalusian School of Public Health, Granada, Spain; 9 Consortium for Biomedical Research in Epidemiology and Public Health (CIBERESP), Madrid, Spain; 10 Public Health Department of Gipuzkoa, Basque Government, Gipuzkoa, Spain; 11 Department of Epidemiology, Murcia Regional Health Authority, Murcia, Spain; 12 Navarre Public Health Institute, Pamplona, Spain; 13 University of Cambridge School of Clinical Medicine, Cambridge, United Kingdom; 14 MRC Epidemiology Unit, Cambridge, United Kingdom; 15 Cancer Epidemiology Unit, Nuffield Department of Clinical Medicine, University of Oxford, Oxford, United Kingdom; 16 WHO Collaborating Center for Food and Nutrition Policies, Department of Hygiene, Epidemiology and Medical Statistics, University of Athens Medical School, Athens, Greece; 17 Department of Epidemiology, Harvard School of Public Health, Boston MA, USA; 18 Hellenic Health Foundation, Athens, Greece; 19 Bureau of Epidemiologic Research, Academy of Athens, Athens, Greece; 20 Molecular and Nutritional Epidemiology Unit, Cancer Research and Prevention Institute – ISPO, Florence, Italy; 21 Fondazione IRCCS Istituto Nazionale dei Tumori, Milan, Italy; 22 Cancer Registry and Histopathology Unit, “Civile - M.P.Arezzo” Hospital, ASP 7, Ragusa, Italy; 23 Center for Cancer Prevention (CPO-Piemonte), Turin, Italy; 24 Human Genetic Foundation (HuGeF), Turin, Italy; 25 Centre for Nutrition and Health (CVG), National Institute for Public Health and the Environment, Bilthoven, The Netherlands; 26 Dept of Public Health and Clinical Medicine, Umeå University, Umeå, Sweden; 27 International Agency for Research on Cancer, Lyon, France; 28 Imperial College, London, United Kingdom; 29 INSERM U590, Centre Léon Bérard, Lyon France; Florida International University, United States of America

## Abstract

The mTOR (mammalian target of rapamycin) signal transduction pathway integrates various signals, regulating ribosome biogenesis and protein synthesis as a function of available energy and amino acids, and assuring an appropriate coupling of cellular proliferation with increases in cell size. In addition, recent evidence has pointed to an interplay between the mTOR and p53 pathways. We investigated the genetic variability of 67 key genes in the mTOR pathway and in genes of the p53 pathway which interact with mTOR. We tested the association of 1,084 tagging SNPs with prostate cancer risk in a study of 815 prostate cancer cases and 1,266 controls nested within the European Prospective Investigation into Cancer and Nutrition (EPIC). We chose the SNPs (n = 11) with the strongest association with risk (p<0.01) and sought to replicate their association in an additional series of 838 prostate cancer cases and 943 controls from EPIC. In the joint analysis of first and second phase two SNPs of the *PRKCI* gene showed an association with risk of prostate cancer (OR_allele_ = 0.85, 95% CI 0.78–0.94, p = 1.3×10^−3^ for rs546950 and OR_allele_ = 0.84, 95% CI 0.76–0.93, p = 5.6×10^−4^ for rs4955720). We confirmed this in a meta-analysis using as replication set the data from the second phase of our study jointly with the first phase of the Cancer Genetic Markers of Susceptibility (CGEMS) project. In conclusion, we found an association with prostate cancer risk for two SNPs belonging to *PRKCI*, a gene which is frequently overexpressed in various neoplasms, including prostate cancer.

## Introduction

Within the prostate tissue, tumor-promoting effects of endogenous hormones and growth factors are thought to be associated with the stimulation of cellular growth and mitosis, and inhibition of apoptosis. In addition to signaling by IGF-I (but also insulin, and other growth factors), the growth and proliferation of cells are co-determined by amounts of energy and essential amino acids available to the cell [1], [2], .

Recent studies have showed that the mTOR (mammalian target of rapamycin) signal transduction pathway integrates these various signals, regulating ribosome biogenesis and protein synthesis as a function of available energy and amino acids, and assuring an appropriate coupling of cellular proliferation with increases in cell size [1], [2]. The mTOR pathway is regulated through a cascade of enzymatic phosphorylation reactions through phosphatidyl-inositol-triphosphate kinase (PI3K)/protein kinase B (PKB-AKT1), atypical protein kinase C (aPKC), AMP-activated protein kinase (AMPK), hamartin/tuberin (encoded respectively by the tuberous sclerosis complex-1 (*TSC1*) and 2 (*TSC2*) genes), ras-homologue enriched in brain (Rheb), regulatory associated protein with mTOR (raptor), and mammalian target of rapamycin (mTOR). mTOR activation in turn leads to phosphorylation of downstream elements that directly control ribosome biogenesis and ribosomal mRNA translation for protein synthesis[4], [5], [6], [7], [8], [9], [10], [11], [12], [13], [14], [15], [16]. Supplementary figure S1 shows a simplified scheme of the mTOR pathway.

This pathway includes several established proto-oncogenes (*PI3K, AKT1*) and tumor suppressor genes (*PTEN* – which reduces mTOR activity through inhibition of *PI3K/AKT1 – TSC1, TSC2*). These genes are often mutated or aberrantly expressed in human malignancies, including prostate tumors[9], [17], [18], [19], [20], [21], [22], [23].

In addition, recent evidence has pointed to an interesting interplay between the mTOR and p53 pathways (reviewed by Levine et al., 2006) [24]. There are two major connections between these pathways, leading to altered response to stress signals after activation of p53, through activation of AMPK, TSC2 and a p53 phosphatase, composed of an alpha-4 subunit and the PP2A catalytic subunit.

We hypothesized that genes in the mTOR pathway and genes of the p53 pathway that directly relate to mTOR may be centrally implicated in prostate carcinogenesis, and that polymorphic alleles in these genes could modify their expression or activity, thus conferring altered prostate cancer susceptibility. SNPs in genes belonging to the mTOR pathway have already been studied in relation to cancer risk, with some promising results [25], [26], [27].

In this report we investigated the genetic variability of 67 key genes in the above mentioned pathways. We tested the association of 1,084 tagging SNPs with prostate cancer risk in a case-control study nested within the European Prospective Investigation into Cancer and Nutrition (EPIC). To our knowledge this is the first report on polymorphisms of these genes and prostate cancer risk.

## Results

Summary characteristics of the study populations are shown in table 1. In the first phase of this study we analyzed 1,084 SNPs in 67 genes involved in the mTOR pathway (as summarized in supplementary table S1) in 815 prostate cancer cases and 1,266 matched controls. We replicated the best hits in an independent population consisting of 838 prostate cancer cases and 943 matched controls.

**Table 1:** Characteristics of the study populations.

Phase I	Controls	Cases
	1,266	815
**Age at recruitment** (Median, Mean,Std)	60.5 (61.3,6.1)	60.4 (60.7,5.8)
**Severity of disease** a		
Non-aggressive	-	657
Aggressive	-	158

Phase II	Controls	Cases
	943	838
**Age at recruitment** (Median, Mean,Std)	59.9 (59.3, 6.3)	59.8 (59.1, 6.5)
**Severity of disease** a		
Non-aggressive	-	748
Aggressive	-	90

a Disease aggressiveness was defined as extraprostatic extension (stage C/D) or high histologic grade (Gleason score ≥8).

### Genotyping success rates and quality control

We had 1,163 SNPs on our GoldenGate array, of which 30 were included as quality controls and 1,133 were in the candidate gene regions of interest in this study. Thirty-four SNPs were dropped because they had a call rate lower than 75%, which is usually indicative of poor genotyping quality. Eleven SNPs (1% of the total) showed strong departure from Hardy-Weinberg Equilibrium (p<10^−5^) and were thus not analyzed further. Four SNPs were monomorphic in this population. This left a total of 1,084 SNPs (96% of those selected initially) in the 67 candidate genes to be analyzed.

The average call rate of the 1,084 SNPs used for statistical analysis was 99.8% (range 85.2%–100%).

Thirty SNPs were included, which had previously been genotyped on the same samples in the context of a different study. The concordance of the new genotypes with the old genotypes was 100%.

We initially included 2,099 samples, and after removing subjects samples with a call rate lower than 75% (n = 39), we had a dataset including 815 prostate cancer cases and 1,239 controls. The incidence density sampling led to duplicate selection of 27 controls so that 1,266 controls were included in the conditional analyses.

Random duplicate samples (∼5%) were also included and concordance of their genotypes was 100.0%.

### Main effects of genotyped SNPs

Eleven SNPs were significantly associated with prostate cancer risk, at a threshold of p<0.01 (p_trend_<0.01 or p_2df_<0.01) (rs520820 in *GADD45A*; rs546950 and rs4955720 in *PRKCI*; rs706711, rs13156223 and rs831123 in *PIK3R1*; rs6797860 in*TP63*; rs11763144 in *PRKAG2*; rs388372 in *RPS6KA2*; rs13337626 in *TSC2*; rs3783501 in *GADD45B*). Supplementary table S2 shows detailed results for all 1,084 SNPs.

### Replication

We genotyped the eleven SNPs from the first phase in an additional set of 838 prostate cancer cases and 943 matched controls. In the second phase SNP rs546950 in the *PRKCI* gene showed a statistically significant association with prostate cancer risk, at the conventional threshold of p<0.05 (p_2df_ = 0.02).

When we analyzed jointly the results from the two sample sets, both *PRKCI* SNPs showed an association with risk (OR_allele_ = 0.84, 95% CI = 0.76–0.93, p_2df_ = 0.0028, p_trend_ = 0.0007 for rs4955720; OR_allele_ = 0.86, 95% CI = 0.78–0.95, p_2df_ = 0.0014, p_trend_ = 0.0020 for rs546950). Results for the first phase, the replication set and for the joint analysis are shown in table 2.

**Table 2:** SNPs genotyped in the first and second phase of the project.

Gene	Study	Cases/Controls		OR_het_(95%CI)^b^	OR_hom_(95%CI)^b^	OR_allele_(95%CI)^b^	p_2df_	p_trend_
rs number	phase	AA/AB/BB^a^	AA/AB/BB^a^					
*GADD45A* c	1	500/259/32	690/423/69	0.82 (0.67–1.01)	0.60 (0.39–0.94)	0.80 (0.68–0.94)	0.0274	0.0079
rs520820	2	432/277/29	427/267/44	1.03 (0.83–1.27)	0.64 (0.39–1.05)	0.93 (0.78–1.10)	0.1859	0.3784
	1+2	938/539/61	1124/692/113	0.92 (0.79–1.06)	0.62 (0.45–0.87)	0.86 (0.76–0.97)	0.0156	0.0117
*GADD45B*	1	181/432/167	349/576/241	1.47 (1.18–1.84)	1.32 (1.01–1.74)	1.16 (1.02–1.33)	0.0033	0.0253
rs3783501	2	213/350/178	219/361/161	1.00 (0.79–1.26)	1.13 (0.86–1.50)	1.06 (0.92–1.22)	0.5853	0.4163
	1+2	397/786/347	570/945/402	1.22 (1.04–1.43)	1.23 (1.01–1.49)	1.11 (1.01–1.23)	0.0368	0.0273
*PIK3R1*	1	475/258/57	708/424/48	0.94 (0.77–1.15)	1.82 (1.21–2.72)	1.13 (0.96–1.32)	0.0073	0.1365
rs13156223	2	397/279/46	416/270/36	1.07 (0.87–1.32)	1.32 (0.84–2.07)	1.11 (0.94–1.31)	0.4275	0.2245
	1+2	876/542/103	1132/695/84	1.01 (0.88–1.17)	1.59 (1.18–2.15)	1.12 (1.00–1.26)	0.0096	0.0423
*PIK3R1*	1	261/356/174	393/608/181	0.92 (0.75–1.14)	1.52 (1.16–1.99)	1.19 (1.04–1.36)	0.0004	0.0105
rs706711	2	223/362/149	235/364/135	1.05 (0.83–1.32)	1.17 (0.87–1.59)	1.08 (0.93–1.25)	0.5901	0.3272
	1+2	485/725/324	631/975/319	0.99 (0.84–1.15)	1.35 (1.11–1.65)	1.14 (1.03–1.26)	0.0026	0.0107
*PIK3R1*	1	660/118/13	942/232/8	0.74 (0.58–0.94)	2.29 (0.89–5.90)	0.85 (0.68–1.06)	0.0082	0.1393
rs831123	2	582/117/7	582/116/8	1.01 (0.76–1.33)	0.88 (0.32–2.42)	0.99 (0.77–1.28)	0.9660	0.9489
	1+2	1250/236/20	1529/352/16	0.83 (0.70–1.00)	1.48 (0.74–2.94)	0.90 (0.76–1.06)	0.0636	0.1994
*PRKAG2*	1	611/163/17	838/314/30	0.72 (0.58–0.89)	0.77 (0.42–1.41)	0.77 (0.64–0.92)	0.0101	0.0047
rs11763144	2	559/174/8	551/177/13	0.97 (0.76–1.23)	0.61 (0.25–1.48)	0.93 (0.75–1.15)	0.5400	0.4781
	1+2	1178/338/25	1397/492/43	0.82 (0.70–0.97)	0.72 (0.44–1.19)	0.83 (0.72–0.95)	0.0310	0.0086
*PRKCI*	1	339/356/96	421/568/193	0.77 (0.63–0.93)	0.62 (0.47–0.83)	0.78 (0.68–0.90)	0.0017	0.0004
rs4955720	2	272/332/109	247/349/117	0.86 (0.68–1.09)	0.85 (0.62–1.16)	0.91 (0.78–1.06)	0.3768	0.2078
	1+2	613/694/206	675/917/312	0.82 (0.70–0.95)	0.72 (0.58–0.89)	0.84 (0.76–0.93)	0.0028	0.0007
*PRKCI*	1	278/384/129	347/588/246	0.79 (0.65–0.98)	0.66 (0.50–0.86)	0.81 (0.71–0.92)	0.0068	0.0017
rs546950	2	250/333/156	207/384/148	0.70 (0.55–0.90)	0.85 (0.63–1.15)	0.91 (0.78–1.05)	0.0160	0.1898
	1+2	528/723/288	558/975/396	0.76 (0.65–0.89)	0.76 (0.62–0.92)	0.86 (0.78–0.95)	0.0014	0.0020
*RPS6KA2*	1	263/419/109	481/527/174	1.48 (1.20–1.82)	1.17 (0.87–1.56)	1.16 (1.01–1.33)	0.0011	0.0317
rs388372	2	264/330/80	264/322/88	1.02 (0.81–1.29)	0.91 (0.64–1.29)	0.97 (0.83–1.14)	0.7920	0.7419
	1+2	531/754/189	748/854/263	1.25 (1.07–1.46)	1.05 (0.84–1.31)	1.08 (0.97–1.19)	0.0120	0.1674
*TP63*	1	356/340/43	561/442/101	1.21 (0.98–1.48)	0.65 (0.44–0.96)	0.97 (0.83–1.13)	0.0051	0.6897
rs6797860	2	377/301/50	376/294/58	1.02 (0.82–1.27)	0.86 (0.57–1.29)	0.97 (0.82–1.14)	0.7161	0.7040
	1+2	740/648/96	947/746/159	1.11 (0.96–1.29)	0.77 (0.58–1.01)	0.97 (0.87–1.09)	0.0238	0.6576
*TSC2*	1	650/133/5	1029/144/5	1.49 (1.15–1.94)	1.62 (0.46–5.68)	1.46 (1.14–1.87)	0.0090	0.0024
rs13337626	2	629/110/4	612/122/9	0.88 (0.67–1.17)	0.44 (0.14–1.43)	0.84 (0.65–1.08)	0.2756	0.1650
	1+2	1289/243/9	1649/268/14	1.15 (0.95–1.39)	0.77 (0.33–1.80)	1.10 (0.93–1.31)	0.2829	0.2767

a AA = homozygotes for the common allele; AB = heterozygotes; BB = homozygotes for the rare allele.

b Results of conditional logistic regression. OR_het_ = odds ratios for the heterozygotes vs. the homozygotes for the common allele; OR_hom_ = odds ratios for the homozygotes for the rare allele vs. the homozygotes for the common allele; OR_allele_ = odds ratios for an increase of one rare allele; 95%CI = 95% confidence interval.

c For each SNP, the first line indicates results of the first phase, second line shows results of the replication phase, and third line indicates results of the joint analysis.

We calculated M_eff_ values for each candidate gene separately and for the whole study (by adding the individual gene M_eff_ values; details are shown in supplementary table S3). The pathway-wide M_eff_ was 849. We therefore used a study-wide significance p-threshold of 0.05/849 = 5.9×10^−5^. Using this threshold, no significant associations (p_trend_<5.9.x10^−5^ or p_2df_<5.9×10^−5^) were observed between any of the polymorphisms genotyped and overall prostate cancer risk.

The two SNPs in *PRKCI* were also genotyped in the context of the Cancer Genetic Markers of Susceptibility (CGEMS) project (http://cgems.cancer.gov/), one of the first genome-wide association studies on prostate cancer susceptibility. The associations observed in the first phase of CGEMS (OR_allele_ = 0.85 p_trend_ = 0.0024 for rs4955720, OR_allele_ = 0.94 p_trend_ = 0.089 for rs546950) were similar to those observed in the present report. In a meta-analysis using the unconditional OR-estimate from the data of the second phase of our study jointly with results from CGEMS, the two SNPs showed very similar results as those obtained with the EPIC data alone (OR_allele_ = 0.91, 95% CI 0.83–0.99, p = 0.029 for rs546950 and OR_allele_ = 0.87, 95% CI 0.79–0.95, p = 0.002 for rs4955720). A meta-analysis performed considering the joint data of the first and second phase of our study with results from CGEMS showed essentially the same results (OR_allele_ = 0.91, 95% CI 0.84–0.98, p = 0.019 for rs546950 and OR_allele_ = 0.85, 95% CI 0.78–0.92, p = 0.00016 for rs4955720).

### Effects of genotyped SNPs in subgroups of disease aggressiveness

We analyzed associations of SNPs with prostate cancer risk by grouping cases according to disease aggressiveness, but we did not observe statistically significant (p<0.05) heterogeneity between strata. Results for the eleven SNPs that were genotyped on the complete dataset are shown in supplementary table S4.

## Discussion

The mTOR pathway is implicated in tumor development, and analogues of rapamycin – a natural antibiotic that specifically interferes with mTOR action (via an additional receptor protein) – are showing great promise as potential therapeutic agents for treating certain types of solid tumors [28], [29], [30]. We hypothesized that genes belonging to the mTOR pathway may be centrally implicated in cancer development, including prostate cancer, and that polymorphic alleles of these genes might affect prostate cancer risk.

In this study, we thoroughly captured common genetic variation across 67 genes in the mTOR pathway and to our knowledge, this is the most comprehensive evaluation of common and coding variation in the mTOR pathway genes in relation with prostate cancer risk. We found an association of two SNPs in the *PRKCI* gene, rs546950 and rs4955720, with a decreased risk of prostate cancer. The first SNP showed an association at the first screening, in a replication set and in a meta-analysis of our second phase with data from CGEMS, while rs4955720 showed an association only in the screening set and in the meta-analysis. Since the two SNPs were selected as tagging SNPs they are not in strong LD, however we cannot exclude that they might reflect the same signal due to a moderate underlying LD (r^2^ between the two SNPs is 0.53).

A role of genetic variation in the *PRKCI* gene in prostate cancer aetiology is plausible given that atypical protein kinase C lambda/iota (aPKCλ/ι), encoded by the *PRKCI* gene, is a protein kinase C isozyme, which plays multifunctional roles in cellular maintenance and growth of epithelial cells[31], [32], [33], [34], [35], [36],[37]. One of the physiological functions of aPKCλ/ι is to mediate insulin-induced increases in glucose transport. Insulin regulates glucose transport through phosphatidyl-inositol-triphosphate kinase (PI3K). Distal effectors of PI3K include protein kinase B (PKB/Akt) and aPKC isoforms ζ and λ/ι [38].

PKC isozymes are also involved in cell proliferation, survival, differentiation and apoptosis. Studies on lung, ovary, colon, and breast cancers have demonstrated a relationship between aPKCλ/ι expression and cancer progression and suggest that aPKCλ/ι expression might predict poor survival[21], [22], [23], [39], [40], [41], [42], [43]. There are several reports showing enhanced aPKCλ/ι expression in human prostate cancer tissues, but the relationship between aPKCλ/ι and prostate cancer progression remains unclear[44], [45]. Furthermore experiments using the prostate cancer cell line DU145 revealed that aPKCλ/ι is involved in prostate cancer growth both *in vivo* and *in vitro* [46]. Overexpression of aPKCλ/ι can be explained with an amplification of the *PRCKI* gene, which has been reported in lung and ovarian cancer [22], [23], [39] or the amplification of chromosome 3q including the *PRCKI* gene which has been reported in prostate cancer cell lines [47]. Another possibility is that aPKCλ/ι expression is up-regulated through the transcriptional activation of *PRCKI* promoter [48].

In this report we found that two allelic variants of the *PRKCI* gene were associated at a study-wise significant level with a decreased risk of prostate cancer. rs546950 and rs4955720 are not situated in the coding region of the gene and it is not immediately evident how to relate the genetic variability to the gene function. We searched public databases for any reported functions of the two SNPs but we did not find evidence pointing to a differential gene expression or mRNA stability. No report to date has been published on either SNP in relation to disease suceptibility. We also ran in silico analysis on the possible changes on trasciption binding sites. These analyses predict a differential binding to the alleles of the two SNPs of various transcription factors. In particular MYOD1 is predicted to bind only to the minor allele (A) of rs546950 and POU3F3 to the minor allele (A) of rs4955720. Both transcription factors have pro-differentiation, anti-proliferation effect [49], [50]. The fact that both of them bind to the minor, protective alleles is intriguing, although MYOD1 and POU3F3 have only been reported to exert their function in the muscle and in the brain, respectively. rs546950 is situated in the first intron of *PRKCI*, very close to the beginning of the gene, therefore this is consistent with a possible involvement in the regulation of transcription of the gene. Since the variant allele exerts a protective effect on prostate cancer risk we can hypothesize that it decreases or increases the ability of transcription factors to bind and in such way that results in a decrease of the gene expression, and consequently decreasing the risk of prostate cancer.

It is more difficult to understand the association between rs4955720 and decreased risk of prostate cancer from a biological point of view. However, in the joint analysis the p value of this SNP was the more significant of the two. This SNP is located at the 3′ of the gene, after the end of the last exon, and a possible function is not immediately evident. Also the possible differential binding with transcription factors seems to be less relevant in this case. rs4955720 could be in high linkage disequilibrium (LD) with another SNP that directly affects the transcription and/or function of *PRKCI*. However, all known common *PRKCI* SNPs in high LD with rs4955720 are located in introns of the gene, and do not seem functionally relevant. In conclusion it is difficult to understand the biological mechanism that could explain the association of the two polymorphism with prostate cancer risk and further functional studies are warranted.

SNPs of some of the PI3K genes we investigated here were also studied in the Breast and Prostate Cancer Cohort Consortium (BPC3) (Koutros 2010). In that study, rs7556371, a SNP of *PIK3C2B*, was shown to be associated with increased prostate cancer risk. In our study we genotyped rs4951384, which tags rs7556371 (r^2^ = 1), but we did not observe any evidence of association (supplementary table S2). However, it has to be noticed that the increase of risk found by Koutros et al was very modest and could therefore be detected only by a study with a huge sample size, such as BPC3.

The intensive SNP tagging approach used provided a close to exhaustive analysis of possible mono-allelic (main effect) associations of prostate cancer risk with common polymorphic variants known for each of the loci studied. We had sufficient power (0.80 for codominant model in the joint analysis) to detect OR = 1.40 (or OR = 0.71 for associations with a reduced risk) at alpha = 5.9×10^−5^ (study-wide significance p-threshold) for a SNP with a minor allele frequency of 0.30.

Although over 97% of the EPIC subjects are estimated to be of Caucasian origin, differences in allelic frequencies across Europe could in theory cause confounding by population stratification. However, we did not observe major variations in allele frequencies across countries for the SNP studied here (data not shown).

A possible drawback of this work is the large number of tests performed, although several hints suggest a real association: 1) we observed associations (p<0.05) in both stages of the study, 2) after correcting for the number of tests (n = 11) performed in the second phase the association between rs546950 and prostate cancer risk remains significant, 3) the association is biologically plausible and 4) the meta-analysis CGEMS/EPIC (2,743 cases and 3,088 controls) showed very similar results as those obtained with the EPIC data alone (p = 0.002 for rs4955720 and p = 0.029 for rs546950.

In conclusion, we found an association with prostate cancer risk for two SNPs belonging to *PRKCI*, a gene which is frequently overexpressed in various cancers, including prostate cancer. This observation warrants replication in a larger study.

## Materials and Methods

### Ethics Statement

All participants signed an informed written consent. The study was approved by the ethical review boards of the International Agency for Research on Cancer, and of the collaborating institutions responsible for subject recruitment in each of the EPIC recruitment centres.

### The EPIC cohort

A fully detailed description of the EPIC cohort has been published elsewhere [51]. Briefly, EPIC consists of about 370,000 women and 150,000 men, aged 35–69, recruited between 1992 and 2005 in 10 Western European countries.

The vast majority (>97%) of subjects recruited in the EPIC cohort are of European (‘Caucasian’) origin. All EPIC study subjects provided anthropometric measurements (height, weight, and waist and hip circumferences) and extensive, standardized questionnaire information about medical history, diet, physical activity, smoking, and other lifestyle factors. About 260,000 women and 140,000 men provided a blood sample.

Cases of cancer occurring after recruitment into the cohort and blood donation are identified through local and national cancer registries in 7 of the 10 countries, and in France, Germany, and Greece by a combination of contacts with national health insurances and/or active follow-up through the study subjects or their next of kin. Follow-up on vital status is achieved through record linkage with mortality registries.

### Selection of case and control subjects

Case subjects were selected among men who developed prostate cancer after blood collection. Control subjects (1–2 controls per case) were selected randomly by incidence density sampling, matching the cases for centre of recruitment, age at blood donation and duration of follow-up. A total of 815 invasive prostate cancer cases and 1,266 controls, for a total of 2,081 subjects, were included in the first phase of the present study. Each control should have been free of cancer up to the duration of follow-up of the index case.

### SNP selection

For each of the 67 candidate genes we selected a genomic region between 5 kb 5′ of the beginning of the first known exon and 5 kb 3′ of the end of the last known exon. A list of SNPs in all 67 gene regions was compiled using data from HapMap (release 22, based on dbSNP version 126 and NCBI genome build 36), and tagging SNPs were selected by use of the Tagger algorithm[52], as implemented in the Haploview software. Parameters used for Tagger selection were minor allele frequency ≥5% in Caucasians, minimum r^2^ = 0.8 between each pair of tagged and tagging SNPs, pairwise tagging (we observed an average mean r^2^ between tagging SNPs and the SNPs they tag of 0.95). SNPs that were predicted to perform poorly with Illumina GoldenGate genotyping technology were either replaced by SNPs in high LD (r^2^ = 0.8, as calculated from HapMap data), or dropped from the list if no proxy was available. This gave us a list of 1,133 SNPs. Finally, for quality control purpose, we added 30 SNPs that had been genotyped on the same samples in an unrelated project.

### Sample preparation and genotyping

DNA was extracted from blood samples on an Autopure instrument (Qiagen, Hilden, Germany) with Puregene chemistry (Qiagen, Hilden, Germany). The order of DNAs from cases and controls was randomized on PCR plates in order to ensure that an equal number of cases and controls could be analyzed simultaneously.

Genotyping was carried out using the Illumina GoldenGate technology (San Diego, CA, USA), according to the protocol specified by the manufacturer.

### Data filtering and statistical analysis

Any sample where greater than 25% of the SNPs failed had all of the SNPs set to missing and these subjects were dropped from analysis. We then filtered data to remove poorly performing SNPs: all SNPs that failed on 25% of samples or more were set to missing, as were all SNPs that showed statistically significant (p<10^−5^) deviations from Hardy-Weinberg equilibrium (HWE) among controls.

We analyzed the association between prostate cancer risk and genotypes for each SNP using conditional logistic regression. Genotypes were coded either as counts of minor alleles (trend test) or as two indicator variables, one for heterozygotes and one for minor-allele homozygotes (two degrees of freedom test).

We performed also analyses in subgroups of disease aggressiveness (aggressive disease was defined as extraprostatic extension (stage C/D) or high histologic grade (Gleason score ≥8).

All statistical analyses were performed using SAS 9.11.

In order to take into account the large number of tests performed in this project, we calculated for each gene the number of effective independent variables, M_eff_, by use of the SNP Spectral Decomposition approach [53]. We obtained a gene-wide M_eff_ value for each gene and also a study-wide M_eff_ value, by adding up the gene M_eff_'s.

### Replication

We replicated the SNPs showing the strongest associations with prostate cancer risk (p_trend_<0.01 or p_2df_<0.01; n = 11) on an additional set of 838 cases and 943 controls selected within the EPIC cohort. All the additional genotyping was carried out using the Taqman assay. The MGB Taqman probes and primers were purchased from Applied Biosystems (Foster City, CA) as pre-designed assays. The reaction mix included 10 ng genomic DNA, 10 pmol each primer, 2 pmol each probe and 2.5 µl of 2x master mix (Applied Biosystems) in a final volume of 5 µl. The thermocycling included 40 cycles with 30 s at 95°C followed by 60 s at 60°C. PCR plates were read on an ABI PRISM 7900HT instrument (Applied Biosystems).

All samples that did not give a reliable result in the first round of genotyping were resubmitted to up to two additional rounds of genotyping. Data points that were still not filled after this procedure were left blank. Repeated quality control genotypes (5% of the total) showed a concordance of 100.0%.

### Bioinformatic analysis

Potential binding sites of transcription factors within the sequence encompassing the two study-wise significantly associated SNPs were performed with MatInspector Professional (http://genomatix.de/cgi-bin/matinspector_prof/mat_fam.pl) [54].

## Supporting Information

Figure S1 Cartoon of the mTOR pathway.
(PNG)

Table S1 Candidate genes and their SNPs.
(DOC)

Table S2 Main effects of 1,084 SNPs genotyped in the first phase. Columns in this table show: gene name, NCBI dbSNP rs number, numbers of cases and controls for each of the three genotypes, odds ratios for heterozygotes, homozygotes for the rare allele (referred to the homozygotes for the common allele) and per allele, with 95% confidence interval, p-value of the test with two indicator variables (2 d.f. test), p-value of the trend test.
(XLS)

Table S3 M_eff_ for each gene in the study.
(XLS)

Table S4 Analyses in subgroups of disease aggressiveness. Columns in this table show: gene name, NCBI dbSNP rs number, possible genotypes, numbers of cases and controls for each of the three genotypes, odds ratios for heterozygotes and for homozygotes for the rare allele (referred to the homozygotes for the common allele), with 95% confidence interval, Cochran's Q parameter, I^2^ statistic, p-value of the test for heterogeneity, p-value of the trend test for each stratum, name of the stratum.
(XLS)
