# Supplementary figures and images for: Genetic Variability of the mTOR Pathway and Prostate Cancer Risk in the European Prospective Investigation on Cancer (EPIC)
Source: PLoS One. 2011 Feb 23;6(2):e16914. doi: 10.1371/journal.pone.0016914 (PMC3044148; doi:10.1371/journal.pone.0016914)

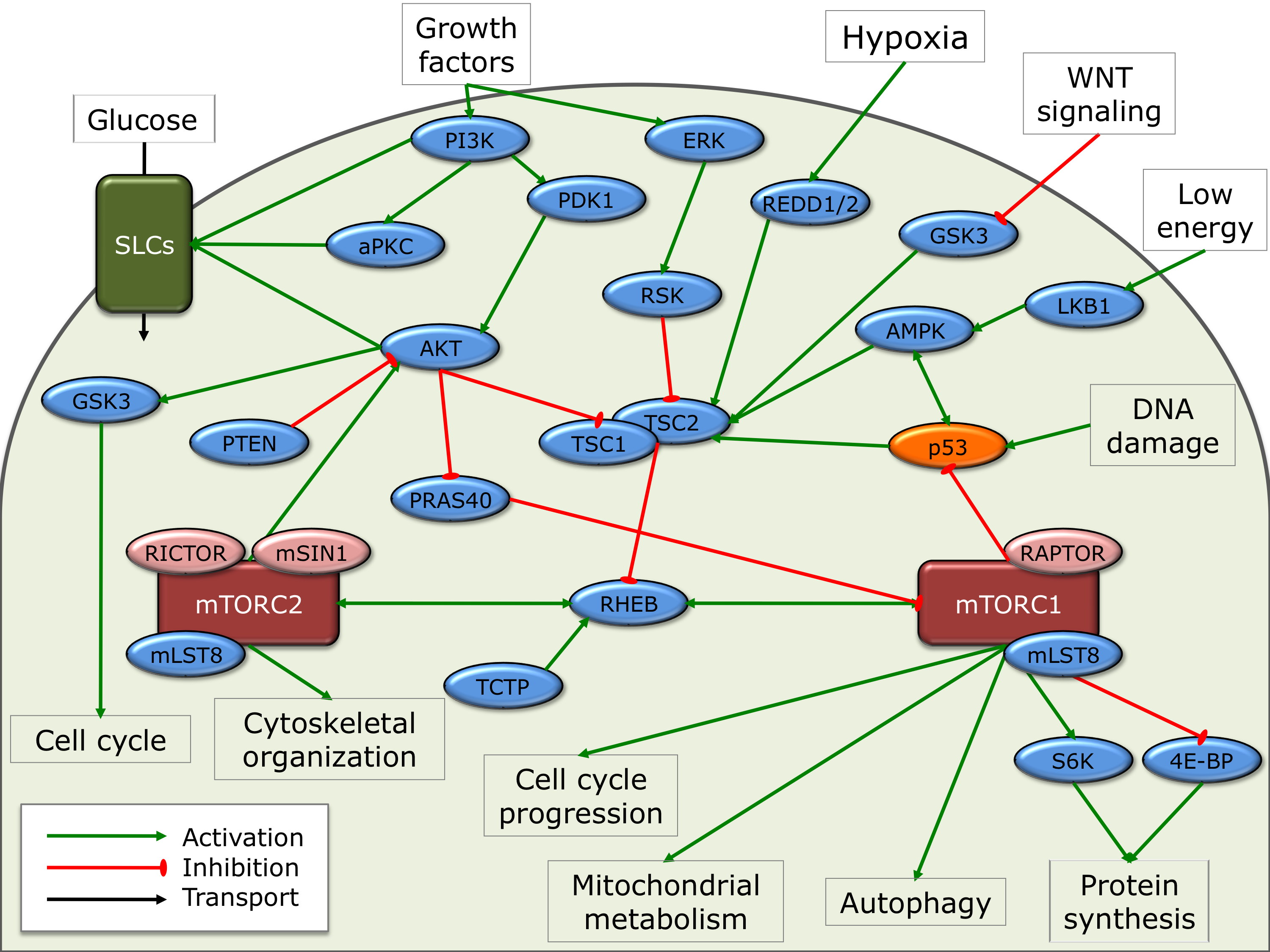

Supplement: Figure S1 — Cartoon of the mTOR pathway. (PNG) [file pone.0016914.s001.png]
