# Supplementary material for: Genetic Variability of the mTOR Pathway and Prostate Cancer Risk in the European Prospective Investigation on Cancer (EPIC)
Source: PLoS One. 2011 Feb 23;6(2):e16914. doi: 10.1371/journal.pone.0016914 (PMC3044148; doi:10.1371/journal.pone.0016914)
Supplement: Table S1 — Candidate genes and their SNPs. (DOC) [file pone.0016914.s002.doc]

**Table S1**. Candidate genes and their SNPs.

| **Gene** | **Trivial name(s)** | **# SNPs MAF≥0.05^a^** | **# tagSNPs r2>0.8^b^** | **# SNPs analyzed^c^** |
| --- | --- | --- | --- | --- |
| **mTOR pathway** | | | | |
| *AKT1* | AKT, PKB | 11 | 5 | 3 |
| *AKT1S1* | PRAS40 | 11 | 3 | 2 |
| *DDIT4* | REDD1 | 5 | 1 | 1 |
| *DDIT4L* | REDD2 | 23 | 11 | 11 |
| *ERBB2* | NEU; NGL; HER2; TKR1; CD340; HER-2; c-erb B2; HER-2/neu | 12 | 5 | 5 |
| *EIF4EBP2* | 4E-BP2 | 10 | 3 | 2 |
| *FRAP1* | mTOR | 41 | 4 | 4 |
| *GBL* | mLST8 | 8 | 3 | 3 |
| *GSK3B* | GSK | 102 | 13 | 10 |
| *KIAA1303* | raptor | 523 | 105 | 94 |
| *MAP4K3* |  | 128 | 15 | 13 |
| *MAPK1* | ERK | 54 | 7 | 6 |
| *MAPKAP1* | mSIN1 | 130 | 16 | 14 |
| *PDPK1* | PDK1 | 3 | 3 | 3 |
| *PIK3C2A* | PI3K | 29 | 5 | 5 |
| *PIK3C2B* | PI3K | 59 | 14 | 12 |
| *PIK3C2G* | PI3K | 358 | 65 | 58 |
| *PIK3C3* | mVps34 | 56 | 5 | 4 |
| *PIK3CA* | PI3K | 38 | 11 | 11 |
| *PIK3CB* | PI3K | 46 | 3 | 2 |
| *PIK3CD* | PI3K | 26 | 14 | 13 |
| *PIK3CG* | PI3K | 29 | 12 | 12 |
| *PIK3R1* | PI3K | 80 | 38 | 33 |
| *PIK3R2* | PI3K | 7 | 5 | 4 |
| *PIK3R3* | PI3K | 63 | 8 | 8 |
| *PIK3R4* | PI3K | 39 | 3 | 3 |
| *PIK3R5* | PI3K | 35 | 17 | 15 |
| *PRKAA1* | AMPK | 29 | 7 | 7 |
| *PRKAA2* | AMPK | 39 | 9 | 9 |
| *PRKAB1* | AMPK | 16 | 3 | 3 |
| *PRKAB2* | AMPK | 26 | 9 | 9 |
| *PRKAG1* | AMPK | 5 | 3 | 3 |
| *PRKAG2* | AMPK | 341 | 146 | 129 |
| *PRKAG3* | AMPK | 3 | 3 | 3 |
| *PRKCI* | DXS1179E, MGC26534, PKCI, nPKC-iota | 21 | 6 | 10 |
| *PTEN* |  | 26 | 9 | 9 |
| *RHEB* |  | 41 | 10 | 10 |
| *RICTOR* |  | 37 | 8 | 7 |
| *RPS6KA1* | p90-RSK | 32 | 14 | 13 |
| *RPS6KA2* | p90-RSK | 686 | 188 | 173 |
| *RPS6KA3* | p90-RSK | 20 | 6 | 6 |
| *RPS6KA4* | p90-RSK | 10 | 4 | 4 |
| *RPS6KA5* | p90-RSK | 125 | 26 | 23 |
| *RPS6KA6* | p90-RSK | 21 | 6 | 6 |
| *RPS6KB1* | S6K, S6K1 | 24 | 8 | 8 |
| *RPS6KB2* | S6K2 | 9 | 3 | 3 |
| *STK11* | LKB1 | 8 | 6 | 4 |
| *TPT1* | hTCTP | 16 | 7 | 6 |
| *TSC1* |  | 54 | 19 | 14 |
| *TSC2* |  | 18 | 11 | 10 |
| **mTOR-p53 pathway** | | | | |
| *PPP2CA* | PP2A | 22 | 2 | 2 |
| *IGBP1* | Alpha 4 | 21 | 7 | 7 |
| *TP53* | p53 | 13 | 7 | 15 |
| *CDKN1A* | p21 | 12 | 5 | 5 |
| *MDM2* |  | 17 | 6 | 6 |
| *TP73* | p73 | 39 | 29 | 25 |
| *TP73L* | p63 | 324 | 112 | 101 |
| *ATM* |  | 69 | 11 | 9 |
| *CHEK2* |  | 33 | 13 | 12 |
| *GADD45A* | GADD45 alpha | 14 | 7 | 7 |
| *GADD45B* | GADD45 beta | 8 | 6 | 4 |
| *GADD45G* | GADD45 gamma | 15 | 4 | 4 |
| *EP300* | p300 | 42 | 7 | 7 |
| *KAT2B* | *PCAF* | 133 | 48 | 42 |
| *ARNT* | HIF-1 beta, aryl hydrocarbon receptor nuclear translocator | 19 | 7 | 7 |
| *TP53BP1* | p53 binding protein 1 | 57 | 4 | 6 |
| *TP53BP2* | p53 binding protein 2 | 47 | 9 | 9 |
| **Total** |  | **4418** | **1178** | **1084** |

^a^ SNPs selected from HapMap release 22.

^b^ tagging SNPs in the selected genes.

^c^ SNPs succesfully genotyped after exclusions and used for statistical analysis.
